# Highly Accurate and Fast Electrochemical Detection of Scrub Typhus DNA via a Nanoflower NiFe-Based Biosensor

**DOI:** 10.3390/bios11070207

**Published:** 2021-06-24

**Authors:** Fengzhen Li, Delun Chen, Wang He, Juan Peng, Yang Cao, Jinchun Tu, Xiaohong Wang

**Affiliations:** 1 Key Laboratory of Advanced Materials of Tropical Island Resources, State Key Laboratory of Marine Resource Utilization in South China Sea, Hainan University, Haikou 570228, China; 18085216210017@hainanu.edu.cn (F.L.); chendelun@hainanu.edu.cn (D.C.); 18085216210012@hainanu.edu.cn (W.H.); cy507@hainanu.edu.cn (Y.C.); tujinchun@hainanu.edu.cn (J.T.); 2 State Key Lab High Efficiency Utilizat Coal & Gre, Natl Demonstrat Ctr Expt Chem Educ, Coll Chem & Chem Engn, Ningxia University, Yinchuan 750021, China; pengjuan@nxu.edu.cn; 3 Key Laboratory of Child Cognition & Behavior Development of Hainan Province, Qiongtai Normal University, Haikou 571127, China

**Keywords:** Scrub typhus, DNA biosensor, NiFe layered double hydroxide, competitive hybridization reaction

## Abstract

Owing to the lack of specific diagnostic methods, Scrub typhus can sometimes be difficult to diagnose in the Asia-Pacific region. Therefore, an efficient and rapid detection method urgently needs to be developed. Based on competitive single-stranded DNA over modified glassy carbon electrode (GCE), an electrochemical biosensor was established to detect the disease. The nano-flower NiFe layered double hydroxide (NiFe-LDH) modified GCE has a large specific surface area, which supported a large amount of gold nanoparticles, so that a wide linear detection range from 25 fM to 0.5 μM was obtained. The beacon DNA (B-DNA) with the same sequence as the Scrub typhus DNA (T-DNA), but labeled with methylene blue, was used to construct a competitive relationship. When T-DNA and B-DNA were present on the sensor simultaneously, they would hybridize with probe DNA in a strong competition, and the corresponding electrochemical response signal would be generated via testing. It contributed to reducing tedious experimental procedures and excessive response time, and achieved fast electrochemical detection of DNA. The strategy provides a worthy avenue and possesses promising applications in disease diagnosis.

## 1. Introduction

Scrub typhus is an acute infectious disease caused by *Orientia tsutsugamushi*, which is harmful to human health. This disease is transmitted to humans through chigger mite bites, and it is endemic in the Asia-Pacific region [1]. Therefore, an early detection method is urgently developed to reduce the harm to humans caused by the disease. The commonly used method for specific early molecular detection for *O. tsutsugamushi* is based on polymerase chain reaction (PCR) detection technology [2,3]. This strategy was used to detect the sequences of Sta56 of *O. tsutsugamushi* in the early stage [4]. Serological diagnosis of *O. tsutsugamushi* disease is limited by the complicated operation. Sensitive and selective detection of nucleic acids, biomolecules, and proteins at low physiological levels is substantially important in life sciences [5,6,7].

To date, various biosensing-based methods, such as fluorescence, colorimetry, and electrochemical approaches, have been used to detect diseases [8,9,10]. Among them, electrochemical strategies have attracted increasing attention in the field of genetic diagnosis because of their superior sensitivity, low price, and high efficiency [11]. In the electrochemical detection process, PCR [12], hybrid chain reaction (HCR) [13], roll circle amplification (RCA) [14], and strand displacement amplification (SDA) [15] amplification strategies are usually required to further improve sensitivity. Although the use of these technologies has advantages, most electrochemical biosensors are still limited by the accuracy of DNA determination [16]. Simplifying the tedious operation steps of the experiment is beneficial to avoid false positives [17]. In the past 10 years, competition reaction strategy has been applied to detect proteins, cells, and nucleic acid [18,19,20,21]. High sensitivity and good selectivity were exhibited owing to the avoidance of errors caused by multiple processing of electrochemical biosensors [22].

Nanomaterials have received considerable attention in recent years because of their unique size, shape, composition, and structure-dependent properties [23]. Nickel–iron composite materials have excellent electrocatalytic properties in the field of electrochemistry. The transition-metal-based materials, especially Ni- and Fe-containing oxygen evolution reaction catalysts, were popular [24,25]. A self-templated way for the preparation of NiFe-layered double hydroxide (NiFe-LDH) nanosheets was reported, which enhanced electrocatalytic performance owing to the porous structure and synergistic effects between Ni and Fe [26]. Furthermore, Ni-based LDHs nanosheets have also been reported to possess catalase activity as efficient mimic enzymes in colorimetric determination of glucose and H_2_O_2_ [27,28].

In this work, an easy method was proposed for the fast and efficient electrochemical detection of Scrub typhus DNA (T-DNA). NiFe-LDH and gold (Au) particles are used to modify glassy carbon electrodes (GCEs) to construct an Au/NiFe-LDH/GCE platform. As an electrode substrate, NiFe-LDH contributed to improving the conductivity and specific surface area of GCEs. Such an improvement was preferable for accelerating electron transfer and loading more AuNPs. In addition, the special technology, DNA chains competition reaction, on the modified electrode has the advantages of simplicity and speed, which is beneficial to reduce experimental operation errors and improve efficiency.

## 2. Materials and Methods

### 2.1. Reagents

All oligonucleotides used in this study were obtained from Sangon Biotech Co., Ltd. (Shanghai, China). The oligonucleotide sequences are described in Table 1. NH_4_F, MgCl_2_, and ethylene diamine tetraacetic acid (EDTA) were procured from Shanghai Macklin Biochemical Co., Ltd. (Shanghai, China). HAuCl_4_·4H_2_O was obtained from Kema Biochemical (Tianjin, China). 6-Mercapto-1-hexanol (MCH), tris (2-carboxyethy) phosphine hydrochloride (TCEP), and tris (hydroxymethyl) methyl aminomethane (Tris-base) were obtained from Sigma–Aldrich (St. Louis, USA). Ni(NO_3_)_2_·6H_2_O, Fe(NO_3_)_3_·9H_2_O, CO(NH_2_)_2_, Na_2_HPO_4_, and NaH_2_PO_4_ and NaCl were procured from Guangzhou Chemical Reagent Factory (Guangzhou, China). SnO_2_ transparent conductive glass (FTO, 1 cm × 2 cm) was purchased from Yingkou New Energy Technology (Yingkou, China). Double distilled water was utilized during the experiment. All chemicals were of analytical reagent grade and used directly after purchase.

### 2.2. Preparation of Nanoflower NiFe-LDH

**Figure 1:**
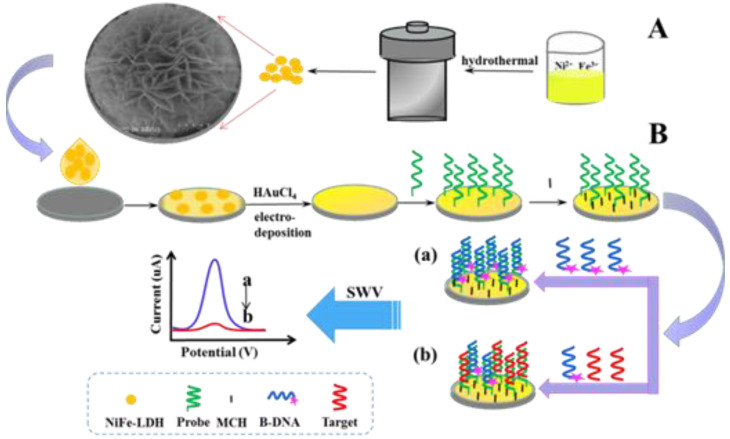
Principle of strand competitive hybridization reaction based on NiFe-LDH for T-DNA electrochemical detection. The process of synthesizing NiFe-LDH (**A**) and the proposed competitive technique (**B**).

NiFe-LDH was prepared according to the previous routine described earlier [29]. Specifically, the parameters were adjusted, and the NiFe-LDH was prepared in solution by self-assembly without any substrate. Firstly, 0.45 mmol Ni(NO_3_)_2_·6H_2_O, 0.15 mmol Fe(NO_3_)_3_·9H_2_O, 3.0 mmol CO(NH_2_)_2_, and 2.0 mmol NH_4_F were put into 21 mL distilled water and stirred until homogeneous. Then, the solution was transferred to a closed autoclave and reacted in an oven at 100 °C for 6 h. Finally, an NiFe-LDH powder was obtained by washing with distilled water and ethanol several times and drying at 60 °C. The process of synthesizing NiFe-LDH is depicted in Figure 1.

### 2.3. Preparation of Modified Electrode

All electrochemical measurements were implemented on an SP-200 Electrochemical Workstation (Paris, France) with a common three-electrode system: platinum wire, saturated Ag/AgCl, and GCE (ɸ = 3 mm) as auxiliary electrode, reference electrode, and working electrode, respectively.

GCE was polished with alumina oxide slurries to gain the mirror surface, then rinsed with ethanol and distilled water, and finally dried. Subsequently, 10 μL of NiFe-LDH suspension (1 mg/mL) was dropped on the GCE surface and dried in room temperature to form a uniform film. Subsequently, AuNPs were deposited on the modified GCE through chronoamperometry method in HAuCl_4_ (1 wt.%) at −0.2 V for 15 s to obtain Au/NiFe-LDH/GCE. The same operation as above was implemented on FTO to obtain Au/NiFe-LDH/FTO.

### 2.4. Fabrication of Competitive Approach and Detection

Prior to P1 immobilization, the disulfide bond of 5′ terminal modified P1 was reduced by incubating it (0.5 μM) with TCEP (25 mM) containing 20 mM of Tris-HCl buffer containing 0.1 M NaCl, 5 mM MgCl_2_, and 1 mM EDTA. Then, 20 μL of diluted P1 was directly incubated onto the Au/NiFe-LDH/GCE at room temperature for 4 h. After washing with 10 mM phosphate buffer containing NaH_2_PO_4_ and Na_2_HPO_4_ (10 mM PBS, pH 7.0), 1 mM MCH was added on the P1/Au/NiFe-LDH/GCE and incubated for 1 h to seal the remaining unbound active sites on AuNPs. The modified electrode was rinsed with PBS again. Then, 20 μL of mixture solution with various concentrations of T-DNA and 0.5 μM competing single-stranded DNA, which was modified with methylene blue (MB) in hydroxy terminal (B-DNA), was added on MCH/P1/Au/NiFe-LDH/GCE and incubated at 50 °C for 10 min. The electrode modified by further washing with PBS was used for electrochemical measurements. The oxidation peak value (I_mix_) produced via SWV test could be easily recorded in PBS. The voltage range was −0.5 to 0 V, with a step potential of 4 mV, a frequency of 25 Hz, and an amplitude of 35 mV. As shown in Figure 1, the proposed competitive technique was conducted on Au/NiFe-LDH/GCE. In the absence of T-DNA, B-DNA was paired with P1, then showing a large number of MB signal molecules produced strong response peaks in SWV. When T-DNA and B-DNA existed simultaneously, the competitive reaction between them and P1 led to a decrease in the number of B-DNA on the modified electrode, and weakening of the electrochemical signal was recorded.

## 3. Results and Discussion

### 3.1. Characterization of Nanomaterials

**Figure 2:**
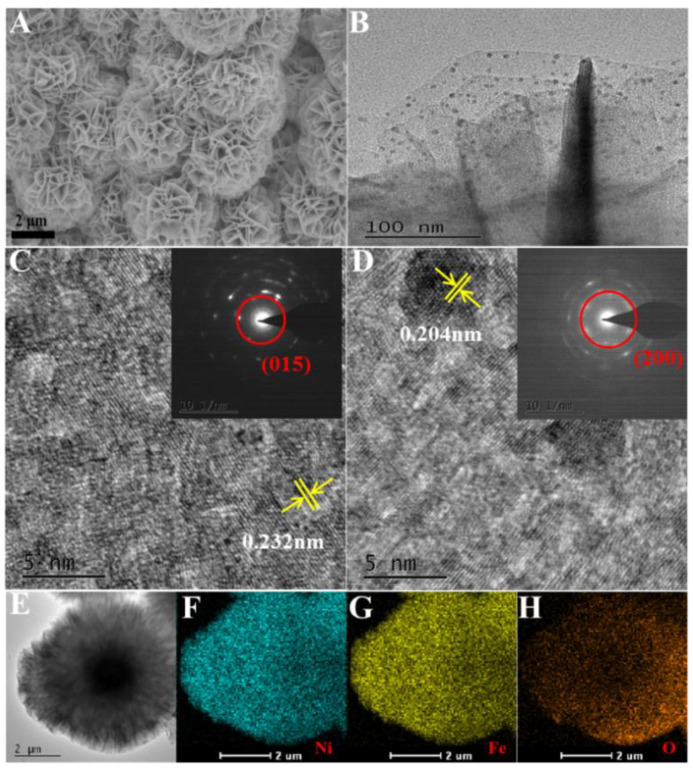
SEM (**A**) of NiFe-LDH, TEM (**B**) of Au/NiFe-LDH/FTO, HRTEM images of NiFe-LDH (**C**), and Au/NiFe-LDH/FTO (**D**). The insets are the corresponding selected area electron diffraction patterns. TEM (**E**) of NiFe-LDH and EDS elemental mapping images (**F**–**H**) of Ni, Fe, and O.

NiFe-LDH was successfully prepared via the classic hydrothermal process. Scanning electron microscopy (SEM) and transmission electron microscopy (TEM) images illustrated that NiFe-LDH presented distinct globular nanoflowers with thin layers (Figure 2A,B). The TEM image displayed some nanoparticles, relatively uniform magnitude and distribution, and anchor on the surface of NiFe-LDH/FTO nanoflowers. The lattice fringes with a distance of 0.232 nm could be matched to the (015) plane of NiFe-LDH (Figure 2C), and the other surrounding lattice fringes with a distance of 0.204 nm, which corresponded to the (200) plane of Au (Figure 2D). Ni, Fe, and O were homogeneously distributed throughout the NiFe-LDH nanoflowers (Figure 2E–H).

**Figure 3:**
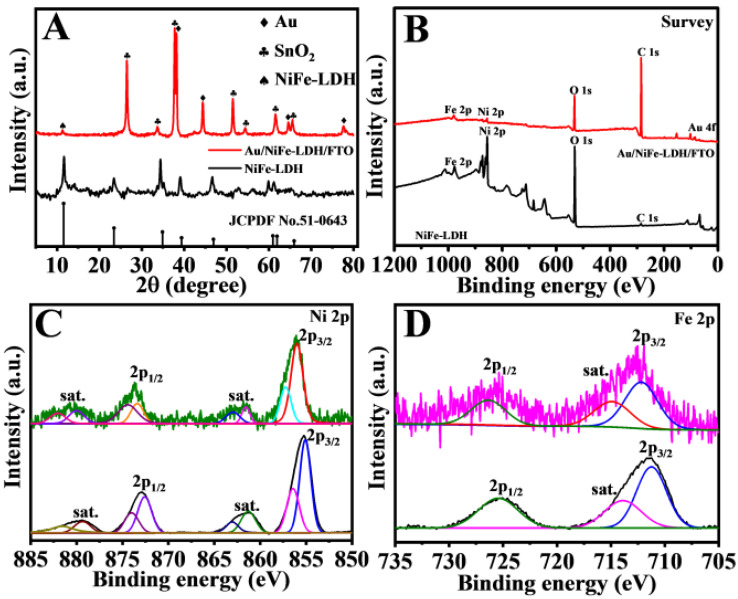
XRD image (**A**), XPS (**B**) survey spectra, Ni 2p (**C**), and Fe 2p (**D**) of Au/NiFe-LDH/FTO and NiFe-LDH.

The NiFe-LDH power was analyzed via X-ray diffraction (XRD), as illustrated in Figure 3A. The peaks at 11.51°, 23.31°, 34.61°, and 39.1° were consistent with the (003), (006), (012), and (015) planes of LDH, respectively, according to PDF number 51-0463 [30].

No other phase demonstrated high-purity LDH. The main peaks at 22.6°, 33.9°, and 51.8° could be attributed to SnO_2_ (PDF number 41-1445). Besides, the diffraction peaks of Au could be found at 38.2°, 44.4°, 64.6°, and 77.5°, which corresponded to the (111), (200), (220), and (311) crystal planes, respectively (PDF number 04-0784). Moreover, the existence of AuNPs was further verified via X-ray photoelectron spectroscopy (XPS).

The chemical components and surface electronic states of NiFe-LDH were observed via XPS (Figure 3B–D). Figure 2B shows the survey spectra demonstrating the existence of C, O, Ni, Fe, and Au. In NiFe-LDH, the high-resolution scan of the Ni 2p^3/2^ peak at 855.7 and 873.4 eV could be assigned to the 2p^3/2^ and 2p^1/2^ of Ni^2+^ (Figure 3C) [31]. With regard to the Fe 2p spectra, the peaks at 711.1 and 723.6 eV could be attributed to Fe 2p^3/2^ and Fe 2p^1/2^, respectively (Figure 3D) [32]. Furthermore, two peaks at around 83.7 and 87.4 eV were observed in the Au 4f spectrum (Figure S1), revealing the existence of AuNPs in the NiFe-LDH samples [33]. No distinct variation to the valence states of Ni and Fe was found after electrodepositing Au on NiFe-LDH.

For the description of the electroactive surface area ($A_{e}$) of the modified electrodes, CV tests were performed at different scanning rates in K_3_[Fe(CN)_6_] solution, which was calculated on the basis of the Randles–Sevcik equation [34].

(1) $$I_{p}=2.69\times 10^{5}A_{e}n^{3/2}D_{0}^{1/2}v^{1/2}C_{0}$$

where $I_{p}$ is the redox peak current; n is the number of electrons participating in the redox reaction, which is taken as 1;$\;D_{0}$ is the diffusion coefficient of the [Fe(CN)_6_]^3−/4−^ species, which is taken as (6.70 ± 0.02) × 10^−6^ cm^2^ s^−1^; v is the scan rate; and $C_{0}$ is concentration of the target molecules in the solution, which is taken as 5 mM.

By calculation, the electroactive surface area of Au/NiFe-LDH/GCE (0.095 cm^2^) was enlarged by 9.2% compared with that of Au/GCE (0.087 cm^2^) by comparing the slope values, while the electroactive surface area of Au/GCE was only 4.8% higher than that of GCE (0.083 cm^2^).

**Figure 4:**
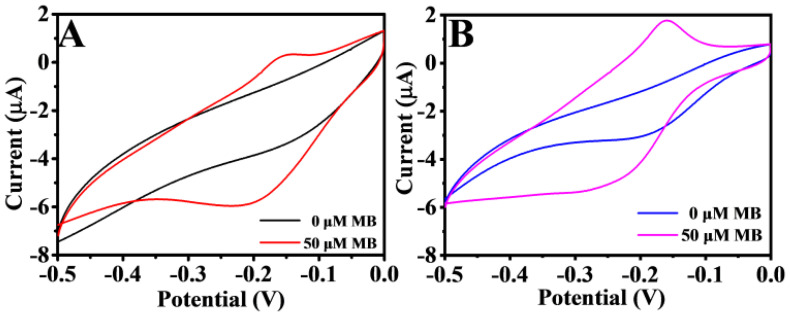
CV of Au/GCE (**A**) and Au/NiFe-LDH/GCE (**B**) in 10 mM PBS (pH 7.0) with 0 and 50 µM MB, respectively. Scan rate: 100 mV/s.

The number of active sites ($n_{a}$) is a critical parameter to explain the electrochemical activity of an electrode or material. It could be estimated via the CVs using the following equation [35,36].

(2) $$n_{a}=Q/2nF\;$$

where Q is the absolute components of the voltammetric charges; F is the Faraday constant, which is taken as 96,485 C/mol; and n is the number of electrons transferred. The CVs of different electrodes are shown in Figure S2. The $n_{a}$ values of Au/NiFe-LDH/GCE, Au/GCE, and GCE were estimated to be 0.783, 0.717, and 0.628 nmol, respectively, based on Equation (2). These results illustrated that the number of active sites increased after modification with Au/NiFe-LDH. Besides, it displayed that the electrochemical activity of the working electrode was strengthened with increased active sites owing to NiFe-LDH providing the large specific surface area, so that AuNPs were widely distributed in the nanoflower structures. NiFe-LDH was verified via CV to further study its catalytic effect on MB. There were different response values of MB at different modified electrodes (Figure 4A,B). At about −0.2 V, the peak oxidation value of 50 μM MB at Au/GCE was 0.57 μA, but that at Au/NiFe-LDH/GCE was 1.15 μA. The response current value of MB is twice as high at Au/NiFe-LDH/GCE as at Au/GCE.

### 3.2. Feasibility of DNA Biosensor

**Figure 5:**
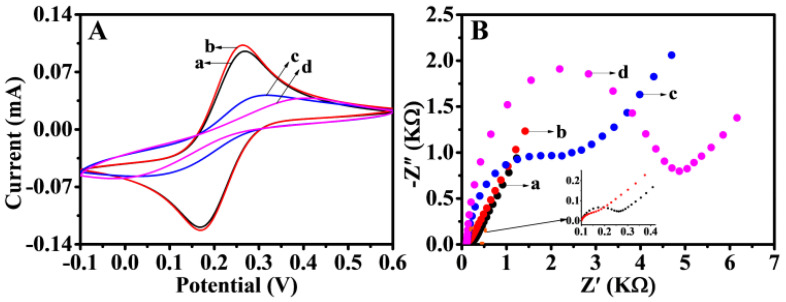
CV (**A**) and EIS (**B**) at bare GCE (a) and Au/NiFe-LDH/GCE (b). P1/Au/NiFe-LDH/GCE (c) and MCH/P1/Au/NiFe-LDH/GCE (d) in 5 mM of K_3_[Fe(CN)_6_] containing 0.1 M KCl.

Typical CV and electrochemical impedance spectroscopy (EIS) tests were executed in 5 mM K_3_[Fe(CN)_6_] solution including 0.1 M KCl. CV was applied to characterize the step-by-step construction of biosensors. The electrochemical behavior of different modified electrodes is shown in Figure 5A. [Fe(CN)_6_]^3−/4−^ obviously showed redox peaks on the bare GCE. After GCE was modified, the oxidation peak increased owing to the NiFe-LDH and AuNPs enhancing the conductivity and specific surface area of GCE, indicating that the materials were successfully immobilized on the working electrode. After P1 was immobilized, the current responses suffered sharp decreases and increased peak separation. The results of slowing down the speed of electron transfer on the electrode surface may be caused by electrostatic repulsion between the negatively charged phosphate backbone of single-strand DNA and [Fe(CN)_6_]^3−/4−^. When the electrode was blocked with MCH, the electrode impedance further increased and the current decreased. This finding may be attributed to MCH occupying the inactive site on the electrode surface, thus hindering the transfer of electrons. EIS could also be applied to study the proposed electrode performance, because the electron transfer resistance value varied with modified programs on GCE (Figure 5B). The EIS and CV results demonstrated a high degree of consistency, and the fabricated biosensor showed a successful assembly process.

The feasibility of the proposed electrochemical detection strategy for T-DNA was also demonstrated. SWV responses of the electrodes prepared step-by-step during the competitive response process were compared (Figure S3). No current was observed with T-DNA/MCH/P1/Au/NiFe-LDH/GCE (curve c), indicating that no MB signaling molecule was attached on the electrode surface. The current of the oxidation peak showed decreased mix-DNA/MCH/P1/Au/NiFe-LDH/GCE (mix-DNA containing B-DNA and T-DNA, curve b). The oxidation current was further enhanced with B-DNA/MCH/P1/Au/NiFe-LDH/GCE (curve a). Such a decrease in the considerable signal response could be ascribed to the competitive reaction.

### 3.3. Optimization of Experimental Conditions

**Figure 6:**
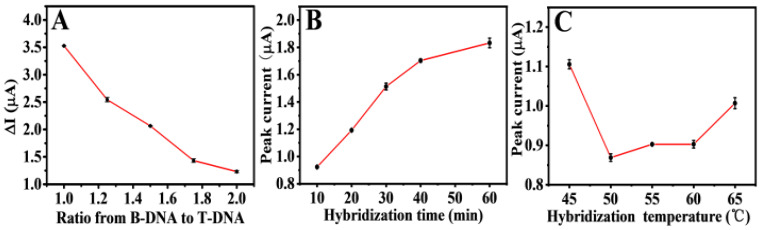
Optimization of the ratio concentration of B-DNA to T-DNA (**A**), hybridization time (**B**), and hybridization temperature (**C**) for the detection of T-DNA on the constructed Au/NiFe-LDH/GCE biosensor. Error bars = SD (n = 3).

Experimental parameters, including the ratio of C-DMA and T-DNA in the mixed solution, hybridization time, and hybridization temperature, were studied to enhance the performance of electrochemical detection. The optimal competitive ratio of B-DNA and T-DNA at the modified electrode was optimized firstly. Figure 6A shows the difference in response currents ∆I (ΔI ($\Delta I=I_{0\;}-{\;I}_{\mathrm{mix}}$, $I_{0\;}$is the peak oxidation produced by 0.5 µM of B-DNA) of various ratios (1:1, 1.25:1, 1.5:1, 1.75:1, and 2:1). The electrode decreased with the increase in B-DNA concentration for 60 min at 55 °C, possibly because the mixed DNA concentration on the electrode surface was too high, resulting in chaotic hybridization of P1, and T-DNA had no strong competitiveness. Thus, 1:1 was used as the optimal ratio.

After the proportion was determined, the hybridization time and hybridization temperature were optimized. As shown in Figure 6B, the larger the oxidation peak, the longer the hybridization time. The reason for this could be that the B-DNA competing chains replaced T-DNA and hybridized with P1 when the hybridization time was over 10 min. The hybridization temperature optimization is exhibited in Figure 6C. At 45 °C, the DNA double helix of 10 min hybridization may be in a metastable state, as the incomplete competitive response simultaneously led to B-DNA and T-DNA strands on P1. Within the temperature range from 55 °C to 60 °C, B-DNA slightly replaced T-DNA, and the competitive hybridization was in a relatively stable plateau stage. The oxidation peak current increased at 65 °C, possibly owing to the weakened competitive hybridization at a higher temperature. T-DNA played the most competitive role at 50 °C, resulting in the minimum amount of B-DNA hybridization on the electrode surface and a low electrochemical response value. Therefore, 50 °C for 10 min was the best condition according to the above optimization.

### 3.4. Sensitivity of Biosensor

**Figure 7:**
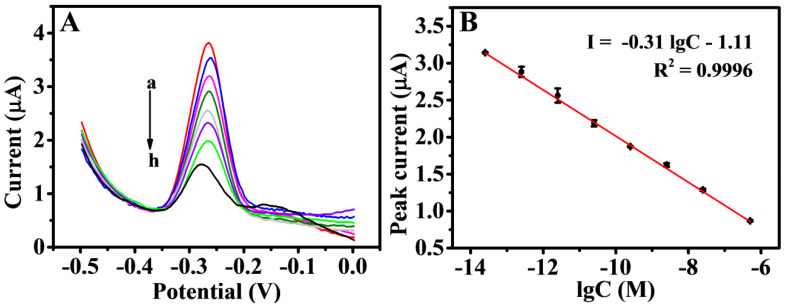
SWV responses (**A**) for various concentrations of T-DNA: 25 fM (a), 250 fM (b), 2.5 pM (c), 25 pM (d), 250 pM (e), 2.5 nM (f), 25 nM (g), and 500 nM (h). Calibration curve (**B**) of peak current versus the logarithm of different concentration of T-DNA. Error bars = SD (n = 3).

On the NiFe-based biosensor, the number of B-DNA was reduced as the concentration of the T-DNA increased. Thus, the SWV response signal of the sensor was reduced accordingly. The cDNA was in the concentration range of 2.5 × 10^–14^–5.0 × 10^–7^ M under the best experimental conditions. The decrease value of the peak current was linearly related to the logarithm of the T-DNA concentration (Figure 7A). The linear regression equation was defined as I (μA) = −0.31 lgC (M) − 1.11, with a correlation coefficient of 0.9996, and the limit of detection (LOD) was 25 fM (S/N = 3) in Figure 7B. This finding showed that the DNA biosensor constructed could achieve quantitative detection of T-DNA. The effective use of NiFe-LDH and the competitive mechanism detection improved the electrochemical response and detection sensitivity of the DNA sensor. The P1 was stably and orderly fixed on the sensor working interface, and the electron transfer rate of the modified electrode was improved. Thus, a simple competitive reaction was realized. As exhibited in Table S2, the detection strategy possessed a wider liner range and relatively lower LOD for T-DNA than several reported electrochemical detections owing to the participation of NiFe-LDH as the signal-enhancing platform and the fewer operational errors in the competitive reaction. The biosensor is single use, and the denaturation step was not performed after each point of the curve calibration.

### 3.5. Selectivity, Reproducibility, and Stability

**Figure 8:**
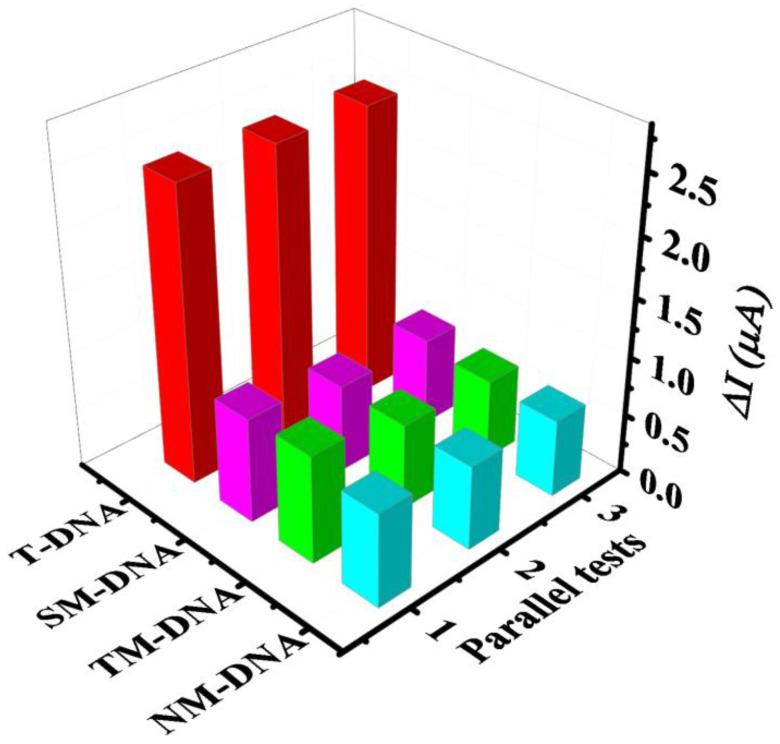
Selectivity of the developed biosensor for T-DNA (500 nM), SM-DNA (500 nM), TM-DNA (500 nM), and NM-DNA (500 nM) throughout SWV.

The selectivity of this proposed electrochemical biosensor with comperirive reaction was investigated by detecting four similar sequences of oligonucleotides, including T-DNA, SM-DNA, TM-DNA, and NM-DNA. SWV was also used to quantitatively analyze the electrochemical response value after hybridization. As shown in Figure 8, the ΔI of T-DNA and B-DNA as the relative intensity was 100%. The intensity of this electrochemical biosensor in the presence of SM-DNA (31.9%), TM-DNA (31.3%), and NM-DNA (29.1%) was lower than that in the presence of T-DNA. Thus, the excellent selectivity of this electrochemical biosensor for T-DNA was proven.

Four modified electrodes were used for the repeated measurement of the electrochemical responses of T-DNA with the competitive strategy to investigate the reproducibility of the developed electrochemical sensor. The relative standard deviation was 3.3%, corresponding to the mixed DNA containing 0.5 μM B-DNA and 25 nM T-DNA. The stability of the biosensor was also evaluated. Four parallel measurement results indicated that the modified electrodes could remain approximately 95.2% of the initial response current value, corresponding to the mixed DNA containing 0.5 μM of B-DNA and 2.5 pM of T-DNA. All the results demonstrated that the prepared DNA sensor possessed good reproducibility and stability.

Concerning the analysis of real samples, we did not discuss this in detail and the reasons are as follows. Firstly, this work focus on giving the novel and simple strategic research for DNA detection, but not building commercial DNA sensors. Then, the feasibility in complex samples is not very important in our topic. Secondly, DNA biosensors are discussed in the references as the methodology and the standard model to analyze the complex samples was not given in previous work. Lastly, PCR was used to obtain the target DNA before the process of concentration detection, so the strategy given in this work could be suitable to the actual sample, theoretically.

## 4. Conclusions

In summary, the NiFe-LDH and AuNPs biosensor was successfully synthetized in a more efficient and green way. The competitive strategy simplifies multiple experimental steps to ensure high accuracy; meanwhile, NiFe-LDH provides good catalytic activity to enhance the electrochemical response of B-DNA. This method exhibited excellent analytical performances in the wider linear range and specificity in electrochemical detection. In addition, the sensitive and high-efficiency electrochemical biosensor has a promising application prospect in other disease DNA detection.

## Figures and Tables

**Table 1:** Oligonucleotide sequences.

Oligonucleotides	Nucleoside Sequences (5′–3′)
Probe DNA (P1)	SH-(CH_2_)_6_-ACCCTATAGTCAATACCAGCA
T-DNA	TGCTGGTATTGACTATAGGGT
Beacon DNA (B-DNA)	TGCTGGTATTGACTATAGGGT-MB
Single-base mismatch DNA (SM-DNA)	TGCTGGTATTAACTATAGGGT
Three-base mismatch DNA (TM-DNA)	TGCTGATATTAACTATATGGT
Non-complementary DNA (N-DNA)	CAAAGCGCTAGCCAGAATCTG

## Supplementary Materials

The following are available online at https://www.mdpi.com/article/10.3390/bios11070207/s1, Figure S1: XPS with deconvolution of Au 4f of Au/NiFe-LDH/FTO; Figure S2: CV at different scanning rates of GCE (A), Au/GCE (B), and Au/NiFe-LDH/GCE (C) in 5 mM K_3_[Fe(CN)_6_] with 0.1 M KCl, and the corresponding fitting curve of GCE, Au/GCE, and Au/NiFe-LDH/GCE (D-F); Figure S3: SWV response of MCH/P1/Au/NiFe-LDH/GCE biosensor in B-DNA with the absence (a) and presence (b) of T-DNA and only the presence of T-DNA (c). The concentration of different DNA all were 5 × 10−7 M. Table S1: Comparison of recently reported electrochemical biosensor for DNA determination.
