# Supplementary material for: Highly Accurate and Fast Electrochemical Detection of Scrub Typhus DNA via a Nanoflower NiFe-Based Biosensor
Source: Biosensors (Basel). 2021 Jun 24;11(7):207. doi: 10.3390/bios11070207 (PMC8301859; doi:10.3390/bios11070207)
Supplement: Supplementary file 1 [file biosensors-11-00207-s001.zip › biosensors-1218874-supplementary.pdf]

Supplementary materials

Highly Accurate and Fast Electrochemical Detection Scrub Typhus DNA via a Nanoflower NiFe-Based Biosensor

Fengzhen Li[a], Delun Chen[a], Wang He[a], Juan Peng[b], Yang Cao[a,c], Jinchun Tu[a], Xiaohong Wang*,[a]

[a] *Key Laboratory of Advanced Materials of Tropical Island Resources, State Key Laboratory of Marine Resource Utilization in South China Sea, Hainan University, Haikou 570228, China.*

E-mail: wangxiaohong@hainanu.edu.cn

[b] *State Key Lab High Efficiency Utilizat Coal & Gre, Natl Demonstrat Ctr Expt Chem Educ, Coll Chem & Chem Engn, Ningxia University, Yinchuan 750021, P. R. China.*

[c] *Key Laboratory of Child Cognition & Behavior Development of Hainan Province, Qiongtai Normal University, Haikou 571127, China.*

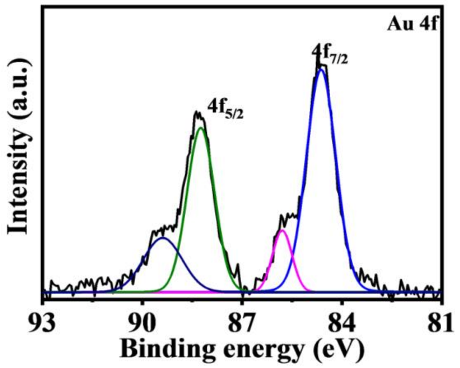

Figure S1. XPS with deconvolution of Au 4f of Au/NiFe-LDH/FTO.

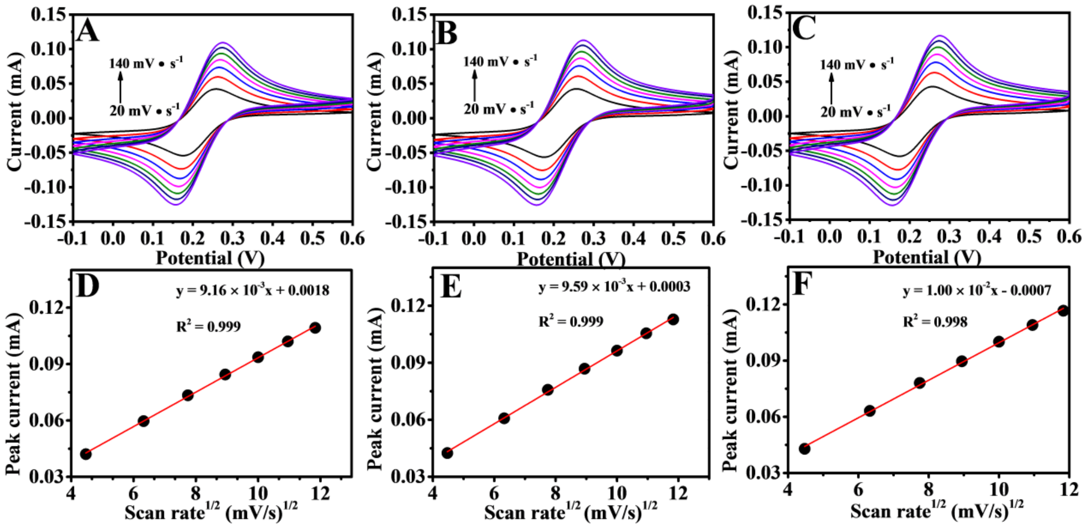

Figure S2. CV at different scanning rates of GCE (A), Au/GCE (B), and Au/NiFe-LDH/GCE (C) in 5 mM $K_3[Fe(CN)_6]$ with 0.1 M KCl, and the corresponding fitting curve of GCE, Au/GCE, and Au/NiFe-LDH/GCE (D-F).

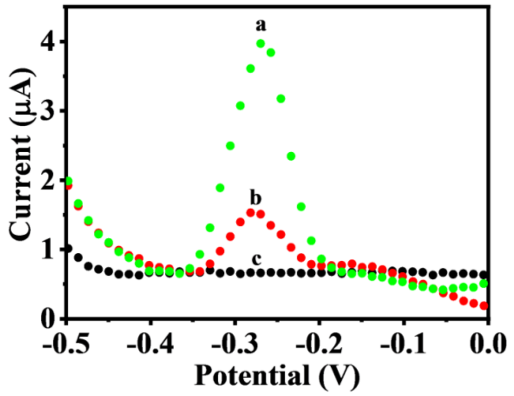

Figure S3. SWV response of MCH/P1/Au/NiFe-LDH/GCE biosensor in B-DNA with the absence (a) and presence (b) of T-DNA and only the presence of T-DNA (c). The concentration of different DNA all were 5×10^{-7} M.

Table S1. Comparison of recently reported electrochemical detection of DNA.

Strategy	Linear range (M)	LOD (M)	Reference
MNAzyme-mediated cycling reaction	1×10^{-13}–1×10^{-8}	2.19×10^{-14}	[1]
AuNPs-GO/GCE biosensor	3.7×10^{-10}–1×10^{-8}	1.6×10^{-10}<br>2.3×10^{-10}	[2]
Proximity hybridization	1×10^{-13}–1×10^{-9}	5.4×10^{-14}	[3]
Toehold strand displacement reaction	1×10^{-12}–1×10^{-7}	2.1×10^{-13}	[4]
AuNPs-PAT/rGO/GCE biosensor	1×10^{-12}–4×10^{-10}	6×10^{-14}	[5]
Catalytic hairpin assembly-programmed	1.5×10^{-8}–2.1×10^{-7}	1.25×10^{-13}	[6]
Competitve strategy on NiFe-LDH sensor	2.5×10^{-14}–5×10^{-7}	2.5×10^{-14}	**This work**

Supplementary references

1. Duan, Y.; Yuan, T.; Xu, Y.; Zhao, M.; Guo, B.; Cheng, W.; Ding, S., Detection of BCR/ABL Fusion Gene Based on MNAzyme-mediated Target-cycling and ssDNA-assisted Cascade Hybridization Reaction. *Electroanalysis* **2018**, 30, (10), 2427-2433.
2. Saeed, A. A.; Sánchez, J. L. A.; O'Sullivan, C. K.; Abbas, M. N., DNA biosensors based on gold nanoparticles-modified graphene oxide for the detection of breast cancer biomarkers for early diagnosis. *Bioelectrochemistry* **2017**, 118, 91-99.
3. Gao, F.; Fan, T.; Wu, J.; Liu, S.; Du, Y.; Yao, Y.; Zhou, F.; Zhang, Y.; Liao, X.; Geng, D., Proximity hybridization triggered hemin/G-quadruplex formation for construction a label-free and signal-on electrochemical DNA sensor. *Biosensors and Bioelectronics* **2017**, 96, 62-67.
4. Hu, Y.; Li, H.; Li, J., A novel electrochemical biosensor for HIV-related DNA detection based on toehold strand displacement reaction and cruciform DNA crystal. *Journal of Electroanalytical Chemistry* **2018**, 822, 66-72.
5. Gholivand, M. B.; Akbari, A., A sensitive electrochemical genosensor for highly specific detection of thalassemia gene. *Biosensors and Bioelectronics* **2019**, 129, 182-188.
6. Wang, M.; Tang, Y.; Chen, Y.; Cao, Y.; Chen, G., Catalytic hairpin assembly-programmed formation of clickable nucleic acids for electrochemical detection of liver cancer related short gene. *Analytica Chimica Acta* **2019**, 1045, 77-84.
